# The Use and Limitations of the 16S rRNA Sequence for Species Classification of *Anaplasma* Samples

**DOI:** 10.3390/microorganisms10030605

**Published:** 2022-03-12

**Authors:** Mitchell T. Caudill, Kelly A. Brayton

**Affiliations:** 1 Program in Genomics, Department of Veterinary Microbiology and Pathology, Washington State University, Pullman, WA 99164, USA; mtcau@vt.edu; 2 Center for One Health Research, Virginia-Maryland College of Veterinary Medicine, Blacksburg, VA 24060, USA

**Keywords:** 16S rRNA, *Anaplasma*, species definition, taxonomy, microbiome

## Abstract

With the advent of cheaper, high-throughput sequencing technologies, the ability to survey biodiversity in previously unexplored niches and geographies has expanded massively. Within *Anaplasma*, a genus containing several intra-hematopoietic pathogens of medical and economic importance, at least 25 new species have been proposed since the last formal taxonomic organization. Given the obligate intracellular nature of these bacteria, none of these proposed species have been able to attain formal standing in the nomenclature per the International Code of Nomenclature of Prokaryotes rules. Many novel species’ proposals use sequence data obtained from targeted or metagenomic PCR studies of only a few genes, most commonly the 16S rRNA gene. We examined the utility of the 16S rRNA gene sequence for discriminating *Anaplasma* samples to the species level. We find that while the genetic diversity of the genus *Anaplasma* appears greater than appreciated in the last organization of the genus, caution must be used when attempting to resolve to a species descriptor from the 16S rRNA gene alone. Specifically, genomically distinct species have similar 16S rRNA gene sequences, especially when only partial amplicons of the 16S rRNA are used. Furthermore, we provide key bases that allow classification of the formally named species of *Anaplasma*.

## 1. Introduction

The genus *Anaplasma* contains obligate intracellular bacteria capable of colonizing both mammalian and arthropod cells. Currently, the genus consists of seven formally named species: *A. marginale*, *A. centrale*, *A. ovis*, *A. bovis*, *A. phagocytophilum*, *A. platys* and *A. caudatum* [1,2]; however, since the last taxonomic organization of the genus [1] at least 25 new species have been proposed in the literature. These proposed species range from clinical isolates of infected animals to unique sequence variants detected in broad multi-species-based 16S rRNA metagenomic studies. Some, such as the proposed “*A. capra*”, represent an important source of human and animal disease [3,4,5], while others appear as single, orphan isolates from unique animal hosts.

The nature and variety of evidence for the claim of a novel species vary greatly in the reports, but a unique 16S rRNA sequence identity appears to be the most prevalent evidence. The intracellular nature of these bacteria and the consequent inability to produce pure cultures make taxonomic studies and new species descriptions in line with the International Code of Nomenclature of Prokaryotes (ICNP) difficult to impossible [6]. Historically, molecular study of the genus has been limited by technical challenges posed by the intracellular lifestyle despite several species being of either public health or economic importance [7,8,9]. The expanded availability of high-throughput sequencing has provided the ability to acquire new genetic samples without the need for associated mammalian or tick-cell culture. While this has provided a boon for the ability to conduct new analyses, the scattershot application of gene sequencing studies lacking a standardized methodology or reference point has led to a proliferation of proposed novel species without the ability to rigorously and systematically analyze these claims en masse. 

Clear species definitions in bacteria are fraught but at the onset of the genomic era of bacterial taxonomy, it was suggested and broadly accepted that bacteria defined as the same species should share at least 70% DNA cross-hybridization (DNA–DNA hybridization or DDH) [10]. DDH assays proved difficult to implement and as genome sequences became available, it was shown that an average nucleotide identity (ANI) of ~95–96% could serve as a proxy for 70% DDH [11]. Following the work of Carl Woese and others, variation in sequence identity of the 16S rRNA gene has been broadly correlated with differences in genomic ANI in prokaryotes, and thus the percentage of DNA–DNA cross-hybridization [12]. While 16S rRNA is a common sequence for metagenomic survey and taxonomic comparison, its utility in resolving isolates to the species level can vary greatly across various groups of bacteria, especially when only partial regions of the genes are used as the point of comparison [13]. In this paper, we evaluate the utility of near-complete and partial 16S rRNA sequences to resolve *Anaplasma* isolates to the species level and test the relationship between 16S rRNA and average nucleotide identity (ANI) for the genus *Anaplasma*. 

We demonstrate that 16S rRNA sequences must be used with caution when attempting to define species within the genus *Anaplasma*, as bona fide species as confirmed by whole genome analysis possess 16S rRNA gene identities suggestive of same species status. Nevertheless, the prevalence of unique 16S rRNA sequences across the literature represents a large measure of uncaptured genetic diversity within the current taxonomy and suggests numerous potential novel species within the genus that deserve deeper geno- and phenotypic characterization. Finally, we present an analysis of the 16S rRNA sequences derived from the complete genomes of the genus to determine key bases that may aid in distinguishing the known, formally named species in the absence of a complete genome.

## 2. Materials and Methods

### 2.1. Sequences and Alignments

All 16S rRNA gene sequences were obtained from NCBI Genbank. Initial searches were conducted using several key phrases including “16S”, “*Anaplasma*”, as well as earlier names such as “*Ehrlichia equi*” that were utilized prior to the reorganization of the order in 2001 [1]. The 737 sequences obtained were compiled and their accession numbers are listed in Supplementary Table S1. A few instances of identical entries were removed, and only sequences over 1300 bp in length were deemed sufficient for inclusion. Additional sequences not captured in the above searches were added following review of the literature for proposed novel *Anaplasma* species. Sequences were aligned utilizing ClustalW within the Bioedit platform (Version 7.0.5.3) [14] according to species designation. Identical sequences were removed to leave a core set of unique 16S rRNA gene sequence variants for each species (see Supplementary Table S2) to construct the similarity tables. Consensus sequences were generated for each species using Bioedit. Ambiguous nucleotides assigned by Bioedit to consensus sequences were examined and kept if the single-nucleotide polymorphism (SNP) was observed in the locus three or more times. A SNP frequency below this threshold was considered within the range of sequence error. A few sequences (AB211164, JQ839010, KP062964–KP062966, AB588977, KX817983, and AF283007) that were classified in Genbank as *A. centrale* were reassigned to *A. capra* (for an explanation, see Khumalo et al., 2018 [15]).

### 2.2. ANI and Sequence Identity Matrix

OrthoANI values were calculated in a pairwise fashion from whole genome sequences using the EZBio platform (https://www.ezbiocloud.net/tools/ani; accessed on 7 March 2022) [16]. The 16S rRNA gene alignments were used to generate sequence identity matrices using the Bioedit platform (Version 7.0.5.3). 

### 2.3. Phylogenetic Comparison 

The phylogenetic tree arising from the consensus 16S rRNA gene sequences was constructed using the Phylogeny.fr platform (www.phylogeny.fr; accessed 7 March 2022) [17,18,19,20,21,22,23]. This platform aligns sequences with MUSCLE (v3.8.31), and removes ambiguous regions with Gblocks (v.091b) using these parameters: block after gap cleaning has minimum length of ten, segments with contiguous non-conserved positions larger than eight are rejected, and flank positions require 85% minimum sequences. The phylogenetic tree was constructed using a maximum likelihood method using PhyML (v3.1/3.0 aLRT) with a HKY85 substitution model with an estimated proportion of invariant sites (of 0.704) and four gamma-distributed rate categories. The gamma shape parameter was estimated directly from the data (gamma = 0.546). Reliability for the internal branch was assessed using the aLRT test (SH-Like). Graphical representation of the phylogenetic tree was performed with TreeDyn (v198.3).

## 3. Results

### 3.1. Correlation of 16S rRNA with ANI within the Genus Anaplasma

An initial analysis was conducted to assess the relationship between 16S rRNA gene sequence identity and genome nucleotide identity within the genus *Anaplasma*. Across the *Anaplasma* species for which genomes are available, sequence similarity in 16S rRNA below 98.7% corresponded to an ANI percentage difference (i.e., < 96%) indicating a distinct species (Table 1). 

This preliminarily analysis confirms the correlation between 16S rRNA identities and ANI for the genus *Anaplasma*. Interestingly, A. *marginale*, *A. centrale*, and *A. ovis* share 16S rRNA sequence identity above 98.7% but nevertheless have distinctive ANI percentages. This indicates that high 16S rRNA sequence similarity may not indicate species status within the genus *Anaplasma*. As expected, within *A. phagocytophilum* and *A. marginale*, the two species for which multiple genomes have been sequenced, there is high intraspecies correlation of both the 16S rRNA identity and the ANI.

### 3.2. 16S rRNA Sequence Similarity across Anaplasma Sequences

Table 2 lists twenty-five proposed species documented in the literature, with the associated NCBI accession numbers of the 16S rRNA sequences. Seventeen of these species had sequences of sufficient length to be used in this study. Of note, none of these proposed species have available whole genome sequences. Additionally, few other genes have been sequenced from these organisms, and a consistent set of genes is not available from each organism to be able to study the utility of a larger gene set for species classification.

Comparison of the 16S rRNA gene sequences from the proposed and validated *Anaplasma* organisms shows that several proposed species have identities greater than the 98.7% cutoff that usually would indicate that two organisms belong to the same species (Table 3). Thirteen of the named species, “*Candidatus* Anaplasma boleense”, ”*Candidatus* Anaplasma pangolinii”, “*Candidatus* Anaplasma testudinis”, *Anaplasma* sp. Saso, *Anaplasma* sp. Hadesa, *Anaplasma* sp. Dedessa, *A. capra*, *A. odocoilei*, *Anaplasma* sp. SA dog, *Anaplasma* sp. ZAM dog, *Anaplasma* sp. Ar. walkerae, *Anaplasma* sp. O. moubata and *Anaplasma* sp. Shizhu, resolve below a 98.7% identity threshold from a formally named species of *Anaplasma*. “*Candidatus* Anaplasma testudinis”, “*Candidatus* Anaplasma pangolinii”, *Anaplasma* sp. Ar. walkerae, *Anaplasma* sp. O. moubata, *Anaplasma* sp. Shizhu and *A. capra* each form independent, distinct, profiles while *Anaplasma* sp. SA dog, and *Anaplasma* sp. ZAM dog group together. *Anaplasma* sp. Saso and *Anaplasma* sp. Hadesa group together as do *Anaplasma* sp. Dedessa and “*Candidatus* Anaplasma boleense”. Interestingly, the putative *A. odocoilei* has less than 98.7% sequence identity with *A. platys*, but greater than 98.7% to other proposed species that group with *A. platys* (i.e., *Anaplasma* sp. Mymensingh, “*Candidatus* Anaplasma camelii”, and *Anaplasma* sp. Omatijenne). In the absence of more sequence data, or a complete genome, it is unknown whether the sequences from these organisms represent truly separate species or are perhaps strain variants *of A. platys*. Notably, two sequences that have been identified as *A. platys* (KU585989 and KU586001) are distinct from the other *A. platys* sequences and may represent yet an additional species.

**Figure 1:**
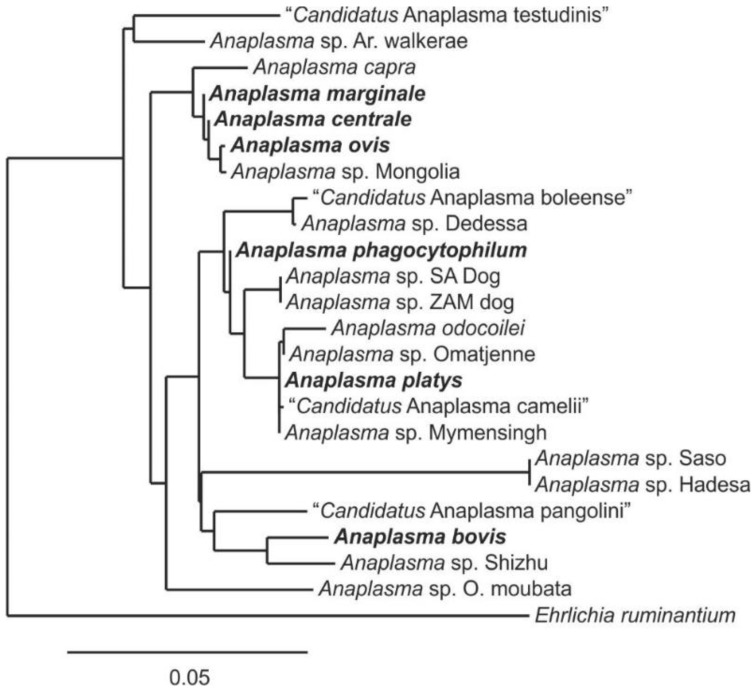
Phylogenetic tree of the genus *Anaplasma* with putative species. Validly named species are represented by their consensus sequences and are highlighted in bold. *Ehrlichia ruminantium* serves as an outgroup for comparison of species distance. Sequences used to construct the tree were 16S rRNA gene regions V2–V7 for each species/putative species and were approximately 1200 bp in length. Phylogeny constructed using a modification of the likelihood-ratio test via the PhyML algorithm with an HKY85 evolutionary model [21,22,23].

**Figure 2:**
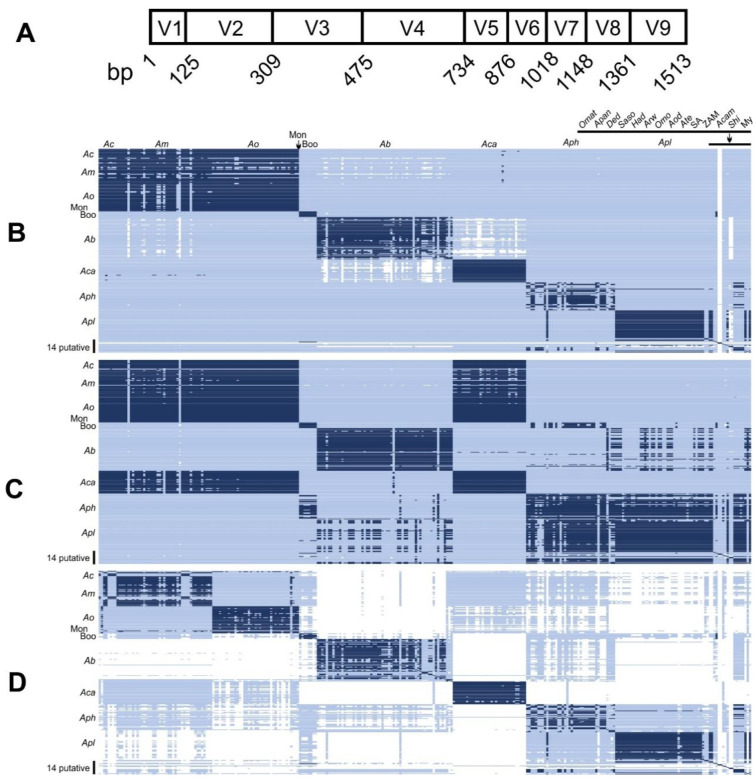
Comparisons of *Anaplasma* 16S rRNA sequences. (**A**) A map of the variable regions of 16S rRNA sequence *A. marginale* St. Maries strain. The start of each variable region was determined by the beginning of the conserved portion of the given variable region. Panels (**B**–**D**) are colorized representations of sequence identity matrices of regions of the 16SrRNA gene for known and putative *Anaplasma* species. (**B**) Comparison of the near-full-length (V2–V9 regions) 16S rRNA sequence identity for 294 sequences of *Anaplasma*. (**C**) Comparison of the V3–V4 regions of 16S rRNA sequence identity 294 *Anaplasma* sequences. (**D**) Comparison of concatenated V2 and V6 regions of 16S rRNA sequence identity 294 *Anaplasma* samples. In (**B**–**D**), each box represents a single 16S rRNA sequence of the indicated species of *Anaplasma*. Dark blue shading represents shared identity above or equal to 98.7% with white representing shared identity below 95%. Light blue shading represents identities between 95–98.7%. Coding is as follows: Ac: *A. centrale*; Am: *A. marginale*; Ao: *A. ovis*; Mon: *Anaplasma* sp. Mongolia; Boo: “*Candidatus* A. boleense”; Ab: *A. bovis*; Aca: *A. capra*; Aph: *A. phagocytophilum*; Apl: *A. platys*; Omat: *Anaplasma* sp. Omatjenne; Apan: “*Candidatus* A. pangolinii”; Ded: *Anaplasma* sp. Dedessa; Saso: *Anaplasma* sp. Saso; Had: *Anaplasma* sp. Hadesa; Arw: *Anaplasma* sp. Ar. walkerae; Omo: *Anaplasma* sp. O. moubata; Aod: *A. odocoilei*; Ate: “*Candidatus* A. testudines”; SA: *Anaplasma* sp. SA dog; ZAM: *Anaplasma* sp. ZAM dog; Acam: “*Candidatus* A. camelii”; Shi: *Anaplasma* sp. Shizhu. My: *Anaplasma* sp. Mymensingh. For explicit sample coding (accession numbers), see Supplementary Table S3.

The relationships among these organisms can be seen in the phylogenetic tree based on 16S rRNA gene sequences (Figure 1). The phylogenetic tree forms two clades, both anchored by three validly named species. The clade anchored by *A. marginale*, *A. centrale*, and *A. ovis* primarily infects erythrocytes. While the other, anchored by *A. bovis*, *A. phagocytophilum* and *A. platys*, primarily infects white blood cells and platelets. To visualize the totality of the sequences used in this study, non-redundant sequences were put into an identity matrix with identities ≥98.7 highlighted in dark blue (Figure 2), with those below 95% in white and intermediate identities in light blue. With this coloration as a guide, several samples appear to not be placed correctly with their most similar counterparts, or they do not appear to have a best fit position in the matrix. In other words, this analysis highlights misplaced organisms/species, such as some *A. marginale* better aligning with *A. capra*. It also shows that some of the putative species, such as “*Candidatus* Anaplasma boleense” and “*Candidatus* Anaplasma testudinis” appear to be unique.

### 3.3. 16S rRNA Variable Region Sequence Analysis across Anaplasma Sequences

Several studies have relied on partial sequences composed of only a few hypervariable regions of the 16S rRNA (see, for instance, [36]) (Figure 2A). As such, we tested the degree to which hypervariable regions corresponded to results arising from the near-complete 16S rRNA sequence. In analyzing these partial sequences, we determined that hypervariable regions 2 and 6 (V2 and V6) were the most variable for *Anaplasma* genotypes. We examined the identity matrices of the near-full-length 16S rRNA sequences (Figure 2B), V3–V4 (Figure 2C) and concatenated V2 and V6 (Figure 2D), to determine the precision and accuracy of specific hypervariable region sequences to classify *Anaplasma* sequences. Using concatenated sequences of V2 and V6 usually resulted in the highest correlation with near-complete sequences (i.e., highest accuracy); however, in a few instances, samples were misclassified into an incorrect species (relatively low precision).

### 3.4. 16S rRNA Single-Nucleotide Polymorphisms across Closely Related Anaplasma Species

Since several distinct species share greater than 98.7% 16S rRNA sequence identity, we also examined the distinct single-nucleotide polymorphisms that might allow classification of a limited sample. In examining the closely related ruminant-infecting *Anaplasma* clade (*A. marginale*, *A. centrale*, *A. ovis* and the proposed species *Anaplasma* sp. Mongolia), only six nucleotides discriminate these species (Table 4). Similarly, when examining the 16S rRNA sequences for the organisms that appear to be closely related to *A. platys*, there are up to thirteen nucleotides that are differentiating, with a smaller number depending on the exact pair/set of sequences being examined (Table 5). Given the lack of genome sequences, an analysis of the ANI relationships could not be performed.

## 4. Discussion

A clear species definition for bacteria has proved a vexing and, indeed, philosophical problem [65,66]. Nevertheless, as a practical matter, the consensus opinion in bacterial taxonomy has been that species should be delineated based on the degree of genetic overlap. The technical test for this genetic overlap has progressed from the degree of DNA–DNA hybridization to average nucleotide identity (ANI), to the percentage of 16S rRNA sequence identity. From the analysis of available sequence samples for the genus *Anaplasma*, we show that classification of samples to the species level remains difficult based on 16S rRNA sequences alone and that ancillary data are likely required to clearly define separate species.

Among the formally named *Anaplasma,* the 16S rRNA sequences from *A. phagocytophilum* are easily distinguishable from those of *A. marginale*, *A. centrale* and *A. ovis*; however, single-nucleotide polymorphisms must be used to distinguish this latter group from each other (Table 4), despite significant whole genome ANI and syntenty differences between these species that confirm their designation as unique entities (Table 1). Conversely, while 16S rRNA sequence comparisons showing identities below a 98.7% threshold are broadly used to suggest that two bacteria are separate species, as shown in Figure 2B for the formally named *Anaplasma* there are numerous sequences in which the samples have been classified as a given species of *Anaplasma* despite the 16S rRNA sequence having less than 98.7% shared identity to a plurality of sequences within that clade. It is possible that these sequences which diverge from the consensus could be novel species erroneously classified as a known species or, alternatively, it may represent a high degree of intraspecies population variance in the 16S rRNA sequence across the *Anaplasma*. Interestingly, the variance appears more prominent in the clade of *Anaplasma* infecting white cells and platelets than the clade infecting erythrocytes. Given the inconsistent sample size of sequence deposits across the various species of *Anaplasma*, and the lack of corroborating information for many deposited sequences, the intraspecies population heterogeneity for the 16S rRNA sequence for each of the *Anaplasma* remains impossible to determine in a rigorous manner, but these tentative findings deserve further investigation.

When the sequence identity of near-complete 16S rRNA sequences from the putative species in the literature are examined, eleven resolve below 98.7% identity with samples from validated species indicating greater diversity within the genus *Anaplasma* than appreciated in the currently accepted taxonomy (Table 3). Several of these also show a low degree of intraspecies variation, though this may result from low sample size and sampling bias in the field study methodology. “*Candidatus* Anaplasma boleense”,”*Candidatus* Anaplasma pangolinii”, “*Candidatus* Anaplasma testudinis”, the two species from argasid ticks and *A. capra* all group as unique entities (Figure 1) and represent likely candidates for independent species. The status and relationships of *A. odocoilei*, *Anaplasma* sp. SA dog, and *Anaplasma* sp. ZAM dog are more complicated. The similarity of 16S rRNA sequences between *A. odocoilei* and several putative species, which themselves have high similarity with *A. platys* may indicate one or even several new species, since 16S rRNA sequence identities above 98.7% can occur among unique *Anaplasma* species. The same is true for *Anaplasma* sp. SA dog and ZAM dog, which group together but are distinct from other *Anaplasma* spp. These may represent variants of a single species (as suggested by Kolo et al., 2020), or two unique entities; additional sequence data or whole genome sequences are needed to resolve this question. *Anaplasma* sp. Dedessa groups with “*Candidatus* Anaplasma boleense” and may represent a variant strain. The same may be true for *Anaplasma* sp. Hadesa and *Anaplasma* sp. Saso, which group together but apart from other *Anaplasma* spp. Collectively, they may represent variance within an independent species. 

The other proposed species for which 16S rRNA sequences are available but do not resolve at a species level in our analysis are *Anaplasma* sp. Mymensingh, “*Candidatus* Anaplasma camelii”, *Anaplasma* sp. Omatjenne and *Anaplasma* sp. Mongolia. *Anaplasma* sp. Mymensingh, “*Candidatus* Anaplasma camelii”, *Anaplasma* sp. Omatjenne all group with *A. platys*, while *Anaplasma* sp. Mongolia groups closely with *A. ovis*. For *Anaplasma* sp. Mymensingh, “*Candidatus* Anaplasma camelii” and *Anaplasma* sp. Mongolia the similarity in 16S rRNA sequence identity with a validly named species were noted in their descriptions and differences in the *groEL* gene sequence was used to justify the creation of a new taxon. For *Anaplasma* sp. Omatjenne [58], the reasoning for novel nomenclature is not clear. The authors noted that *Anaplasma* sp. Omatjenne has 99.5% sequence similarity with *A. platys* but did not elaborate on why they assigned a new name. Presumably it is due to the non-standard host species—*Anaplasma* sp. Omatjenne was found in sheep, while *A. platys* is traditionally found in dogs, but this remains speculative on our part.

Our analysis revealed that sequences covering only a few hypervariable regions of the 16S rRNA gene can lead to misclassification of species (Figure 2) and should be avoided in favor of complete or near-complete 16S rRNA sequences. As such, 16S rRNA sequences alone remain poorly suited for species assignment for the genus *Anaplasma*. This should particularly be noted by those attempting to discriminate *Anaplasma* species when performing microbiome analysis of ticks or other arthropods. Our findings largely comport to recent studies of the use of *groEL* for taxonomic placement and strongly indicate that gene specific knowledge is required for the creation of accurate phylogenies [67]. While *groEL* and *gltA*, along with various *Anaplasma* specific *msp* genes, were additionally considered for our analysis, ultimately too few sequences were available, especially when attempting to match species or strain specific sequences (e.g., pairing a 16S rRNA sequence and *groEL* sequence arising from the same bacterial isolate). There is, at the present time, no rigorous correlation between differences in sequence identity ratios of 16S rRNA and other housekeeping genes within an *Anaplasma* species or among the validly named *Anaplasma* species.

It bears emphasizing that *Anaplasma* species remain difficult to culture in laboratory settings as they require intracellular replication in either mammalian or arthropod cells. This dramatically limits the ability to generate high-quality genetic material suitable for whole genome sequences, particularly for the clinically observed isolates or those arising from metagenomic analyses. While some third-generation sequencing technologies may aid in relieving this barrier, the inability to generate whole genome sequences remains the greatest bottleneck in mapping the diversity of the genus *Anaplasma*.

From our analyses it is clear that genetic diversity in *Anaplasma* is greater than currently captured by the formally named species in the genus *Anaplasma*, but a more definitive description of the exact number and relationship of species requires further genetic and genome sequencing. We would furthermore urge caution in denoting a new species on the basis of only two genes as in the case of *Anaplasma* sp. Mymensingh, “*Candidatus* Anaplasma camelii”, and Anaplasma sp. *Mongolia*, or particularly for naming putative species (i.e., “*Candidatus* Anaplasma corsicanum”, “*Candidatus* Anaplasma ivorensis”, and “*Candidatus* Anaplasma mediterraneum”) on the basis of 23S rRNA sequences, for which there are only a handful of sequences deposited even for validly named *Anaplasma* species. Multilocus sequencing may represent a valuable way forward to overcome some of the current issues in *Anaplasma* taxonomy, as well as a larger number of whole genome sequences for all of the known species of *Anaplasma*. Accessing samples that are known not to be mixed (i.e., containing more than one species of *Anaplasma*) with sufficiently high concentrations of target DNA remains challenging.

## Figures and Tables

**Table 1:** ANI to 16S rRNA Gene Percent Identities for *Anaplasma* species.

	*A. phagocytophilum*				*A. marginale*		*A. centrale*	*A. ovis*
Strain	HZ	HZ2	Norway V2	JM	St. Maries	Florida	Israel	Haibei
HZ		99.98–100%	96.51–99.8%	99.69–100%	67.89–96.3%	68.10–96.3%	68.17–96.3%	68.32–96.2%
HZ2	99.98–100%		96.57–99.8%	99.63–100%	67.78–96.3%	68.38–96.3%	68.20–96.3%	68.22–96.2%
Norway V2	96.51–99.8%	96.57–99.8%		96.43–99.8%	68.16–96.3%	68.44–96.3%	68.26–96.3%	68.13–96.2%
JM	99.69–100%	99.30–100%	96.43–99.8%		68.10–96.3%	68.27–96.3%	67.96–96.3%	67.70–96.2%
St. Maries	67.89–96.3%	67.78–96.3%	68.16–96.3%	68.10–96.3%		99.02–99.9%	87.56–99.3%	84.87–99.3%
Florida	68.1–96.3%	68.38–96.3%	68.44–96.3%	68.27–96.3%	99.02–99.9%		87.81–99.2%	85.28–99.3%
Israel	68.17–96.3%	68.20–96.3%	68.26–96.3%	67.96–96.3%	87.56–99.3%	87.81–99.2%		81.46–99.5%
Haibei	68.32–96.2%	68.22–96.2%	68.13–96.2%	67.70–96.2%	84.87–99.3%	85.28–99.3%	81.46–99.5%	

White text on black background indicates species that conform to the ANI to 16S rRNA gene percent identity for grouping in a species. The black text on a gray background highlights strains that share high 16S rRNA gene identity with another species, but low ANI values. Black text on a white background shows values for which both ANI and 16S percent indicate different species classification.

**Table 2:** Putative *Anaplasma* spp., host source, 16S rRNA sequence accession numbers and references.

Putative Species	Host	Accession #	Ref
“*Candidatus* A. corsicanum”	Sheep	None	[24]
“*Candidatus* A. ivorensis”	Tick (*Amblyomma variegatum*)	None	[25]
“*Candidatus* A. mediterraneum”	Sheep	None	[24]
“*Candidatus* A. africae”	Sheep, Cattle, Goats	MN317253–MN317255 *	[26]
“*Candidatus* A. boleense”	Mosquitos, Cattle	KU585969, KU586025	[27]
		KU586041, KU586162	[27]
		KU586164, KU586169	[27]
		KU586177, KU586180	[27]
		KU586182	[27]
		MH169152 *	[28]
“*Candidatus* A. camelii”	Camels	KX765882	[29]
		KF843823–KF843825	[30]
“*Candidatus* A. rodmosense”	Mosquitos	KU586127 *, KU586148 *	[27]
		KU586144–KU586146 *	[27]
		KU586134–KU586136 *	[27]
		KU586141 *	[27]
“*Candidatus* A. sphenisci”	Penguin (*Spheniscus demersus*)	MG748724 *	[31]
“*Candidatus* A. pangolinii”	Pangolin (*Manis javanica*),	KU189193	[32]
	Tick (*Amblyoma javanense*)	AF497580 *	[33]
“*Candidatus* A. testudines”	Tortise (*Gopherus polyphemus*)	MT62341-MT62345	[34]
“*Cadidatus* A. amazonensis”	Sloths	None	[35]
“*Candidatus* A. brasiliensis”	Anteaters	None	[35]
*A. mesaeterum*	Sheep	None	[36,37]
*A. capra*	Human,	KR261618–KR261622	[38]
	domestic and wild ruminants,	KP314237–KP314238	[38]
	Dogs	KM206273	[39]
		MG869526–MG869594	[3]
		MG869482–MG869510	[3]
		MH762071–MH762077	[40]
		AB211164	[41]
		AB454075	[42]
		AB509223	[43]
		AB588977	[44]
		AF283007	[45]
		EU709493	[46]
		FJ389574, FJ389576	[46]
		JN558820, JN558827	[47]
		KP062964–KP062966	[48]
		KP314241	[38]
		KX817983	[49]
		KX987331	[50]
		LC432092–LC432126	[51]
		MT798599–MT798604	[52]
		MW721591	[53]
*A.* sp. SA dog	Dogs	AY570538–AY570540	[54]
*A.* sp. Mymensingh	Ticks (*Rhipicephalus microplus*;	MF576175.1	[55]
	*Haemaphysalis bispinosa*)	MK815558-MK814449	[56]
*A. odocoilei*	Deer (*Odocoileus virginianus*)	NR_118489, JX876644	[57]
*A.* sp. Omatjenne	Sheep, Cattle, Goats	U54806	[58]
		KC189853	[59]
*A.* sp. Mongolia	Sheep	MK575506	[60]
*A.* sp. ZAM dog	Dogs	LC269823	[61]
*A.* sp. Izard agent	Izard (*Rupricapra pyrenaica*)	EU857675 *	[62]
*A.* sp. Hadesa	Cattle	KY924884	[63]
*A.* sp. Saso	Cattle	KY924885	[63]
*A.* sp. Dedessa	Cattle	KY924886	[63]
*A.* sp. O. moubata	Tick (*Ornithodoros moubata*)	LC558313	[64]
*A.* sp. Ar. walkerae	Tick (*Argas walkerae*)	LC558314	[64]
*A.* sp. Shizhu	Goats	FJ389575	[46]

* These are partial or fragmented sequences that were not included in the analyses. “Accession #” refers to the Genbank sequence accession number.

**Table 3:** Sequence identity matrix for 16S rRNA gene “consensus” sequences for *Anaplasma* spp.

	cent	marg	ovis	Mon	capra	bovis	phag	platys	Mym	Omat	cam	odoc	SA	ZAM	Saso	Hade	bole	Dede	pang	test	walk	moub	Shiz
*A. centrale*	ID	99.7	99.2	99.3	98.1	94.6	95.5	95.7	96.0	95.9	95.9	95.7	95.8	95.9	94.3	94.3	96.2	96.5	96.3	96.4	96.8	96.1	97.2
*A. marginale*	99.7	ID	99.0	99.0	98.1	94.5	95.5	95.8	96.0	95.9	95.9	95.6	95.7	95.8	94.0	94.0	96.1	96.4	96.1	96.4	96.8	96.1	97.0
*A. ovis*	99.2	99.0	ID	99.5	97.8	94.5	95.6	95.6	95.9	95.9	95.9	95.9	95.7	95.8	94.3	94.3	96.0	96.3	96.0	96.5	96.8	96.2	96.9
*A.* sp. Mongolia	99.3	99.0	99.5	ID	97.9	94.6	95.6	95.7	96.0	95.9	95.9	96.0	96.0	96.1	94.4	94.4	96.2	96.5	96.3	96.7	96.8	96.4	97.0
*A. capra*	98.1	98.1	97.8	97.9	ID	93.9	95.2	95.5	95.9	95.5	95.8	95.4	95.7	95.8	93.9	93.9	95.8	96.1	95.6	95.4	96.5	95.9	97.2
*A. bovis*	94.6	94.5	94.5	94.6	93.9	ID	94.9	94.9	95.4	95.1	95.5	95.3	95.4	95.5	92.9	92.9	94.6	94.7	96.4	93.2	94.2	94.5	96.3
*A. phagocytophilum*	95.5	95.5	95.6	95.6	95.2	94.9	ID	96.9	97.0	96.8	97.0	96.8	97.8	97.9	94.0	94.0	96.7	96.9	96.4	94.4	95.5	95.2	95.9
*A. platys*	95.7	95.8	95.6	95.7	95.5	94.9	96.9	ID	99.0	98.9	99.0	98.2	97.2	97.2	93.9	93.9	96.6	96.8	96.4	94.5	95.6	95.2	95.9
*A.* sp. Mymensingh	96.0	96.0	95.9	96.0	95.9	95.4	97.0	99.0	ID	99.5	99.9	98.9	97.9	98.0	94.1	94.1	97.1	97.2	97.0	94.7	96.0	95.7	96.3
*A.* sp. Omatjenne	95.9	95.9	95.9	95.9	95.5	95.1	96.8	98.9	99.5	ID	99.5	98.8	97.5	97.6	94.0	94.0	96.9	97.1	96.7	94.7	95.9	95.4	96.0
*Can* A. camelii	95.9	95.9	95.9	95.9	95.8	95.5	97.0	99.0	99.9	99.5	ID	98.8	97.8	97.9	94.0	94.0	97.0	97.2	96.9	94.8	95.9	95.8	96.3
*A.* odocoilei	95.7	95.6	95.9	96.0	95.4	95.3	96.8	98.2	98.9	98.8	98.8	ID	97.5	97.6	94.1	94.1	96.8	97.0	96.8	94.9	95.9	95.7	96.1
*A.* sp. SA Dog	95.8	95.7	95.7	96.0	95.7	95.4	97.8	97.2	97.9	97.5	97.8	97.5	ID	99.9	94.6	94.6	97.4	97.6	97.2	94.8	95.8	95.8	96.5
*A.* sp. ZAM dog	95.9	95.8	95.8	96.1	95.8	95.5	97.9	97.2	98.0	97.6	97.9	97.6	99.9	ID	94.7	94.7	97.5	97.7	97.2	94.9	95.8	95.9	96.6
*A.* sp. Saso	94.3	94.0	94.3	94.4	93.9	92.9	94.0	93.9	94.1	94.0	94.0	94.1	94.6	94.7	ID	100	94.4	94.5	94.8	93.6	94.0	93.7	94.3
*A.* sp. Hadesa	94.3	94.0	94.3	94.4	93.9	92.9	94.0	93.9	94.1	94.0	94.0	94.1	94.6	94.7	100	ID	94.4	94.5	94.8	93.6	94.0	93.7	94.3
*Can* A. boleense	96.2	96.1	96.0	96.2	95.8	94.6	96.7	96.6	97.1	96.9	97.0	96.8	97.4	97.5	94.4	94.4	ID	99.6	96.6	95.2	96.4	95.3	96.4
*A.* sp. Dedessa	96.5	96.4	96.3	96.5	96.1	94.7	96.9	96.8	97.2	97.1	97.2	97.0	97.6	97.7	94.5	94.5	99.6	ID	96.7	95.4	96.5	95.4	96.7
*Can* A. pangolini	96.3	96.1	96.0	96.3	95.6	96.4	96.4	96.4	97.0	96.7	96.9	96.8	97.2	97.2	94.8	94.8	96.6	96.7	ID	94.7	95.6	96.6	96.5
*Can* A. testudinis	96.4	96.4	96.5	96.7	95.4	93.2	94.4	94.5	94.7	94.7	94.8	94.9	94.8	94.9	93.6	93.6	95.2	95.4	94.7	ID	96.2	95.3	94.9
*A.* sp. Ar. walkerae	96.8	96.8	96.8	96.8	96.5	94.2	95.5	95.6	96.0	95.9	95.9	95.9	95.8	95.8	94.0	94.0	96.4	96.5	95.6	96.2	ID	95.9	96.4
*A.* sp. O. moubata	96.1	96.1	96.2	96.4	95.9	94.5	95.2	95.2	95.7	95.4	95.8	95.7	95.8	95.9	93.7	93.7	95.3	95.4	96.6	95.3	95.9	ID	95.7
*A.* sp. Shizhu	97.2	97.0	96.9	97.0	97.2	96.3	95.9	95.9	96.3	96.0	96.3	96.1	96.5	96.6	94.3	94.3	96.4	96.7	96.5	94.9	96.4	95.7	ID

White text on black background indicates organisms with sequence identity of >98.7%. The species epithet is shown in full on the left side. The species/putative species are listed from left to right with abbreviated names along the top, and full names from top to bottom at the left side in the same order. *Can* = *Candidatus*.

**Table 4:** Differentiating bases for the ruminant clade of *Anaplasma* species.

	Base Number *					
	144	156	220	265	274	1250
*A. centrale*	A	A	T	T	G	T
*A. marginale*	A	G	T	T	G	T
*A. ovis*	G	R	Y	C	T	T
*Anaplasma* sp. Mongolia	G	A	C	C	G	C

* Numbering based on *Anaplasma marginale* St. Maries strain sequence.

**Table 5:** Differentiating bases for *Anaplasma platys* and closely related species.

	Base Number *													
	213	224	262	289	693	696	878	879	885	886	890	1052	1309	1358
*A. platys*	A	T	T	T	N	T	R	C	G	T	T	R	Y	C
*Anaplasma* sp. Mymensingh	A	T	T	T	C	T	A	C	G	T	T	A	C	C
*Anaplasma* sp. Omatjenne	A	C	T	T	C	T	R	C	G	T	T	G	C	T
*“Candidatus* Anaplasma camelii”	A	T	T	T	C	T	A	C	G	T	T	A	T	C
*A. odocoilei*	G	A	G	C	A	A	G	T	A	C	C	G	C	C

* Numbering based on *Anaplasma platys* S3 strain genome sequence. The consensus sequence of *A. platys* contains a “C” between bases at position 555–556 and a “T” between bases at position 1030–1031. These insertions are not present in all *A. platys* strains and are absent in the S3 strain.

## Data Availability

Publicly available data were analyzed in this study. All data were downloaded from NCBI (www.ncbi.nlm.nih.gov/nuccore/) on 17 September 2021. Specific accession numbers are listed in Supplementary Table S1.

## Supplementary Materials

The following supporting information can be downloaded at: https://www.mdpi.com/article/10.3390/microorganisms10030605/s1, Table S1: All 16S rRNA sequence accessions gathered for *Anaplasma*. Table S2: List of the representative sequences used in analysis, and identical sequences that were removed for Figure 2. Table S3: Identity matrices of the 16S rRNA gene regions.
